# Potential targets for boron transport in boron neutron capture therapy

**DOI:** 10.3389/fonc.2026.1817038

**Published:** 2026-05-28

**Authors:** Yucai Wei, Weijing Zhu, Qian Chen, Huda Alnufaei, Yumin Li, Kate Ricketts

**Affiliations:** 1 Cancer Center, The Second Hospital of Lanzhou University, Lanzhou, China; 2 Division of Surgery and Interventional Science, University College London, London, United Kingdom; 3 Key Laboratory of Digestive System Tumor of Gansu Province, The Second Hospital of Lanzhou University, Lanzhou, China; 4 The Second Clinical Medical School, Lanzhou University, Lanzhou, China

**Keywords:** boron delivery, boron neutron capture therapy, boron transporters, molecular targets, tumor-selective accumulation

## Abstract

Boron Neutron Capture Therapy (BNCT) is a binary cancer therapy that involves boron (^10^B) drug administration and epithermal/thermal neutron irradiation. The nuclear fission reaction between low-energy thermal neutrons and ^10^B atoms accumulated in cancer cells generate high linear energy transfer (LET) species (α and ^7^Li particles). These species are short range (4-10 μm, less than a cell diameter) and therefore offer potential to kill cancer cells selectively and spare surrounding healthy cells if the ^10^B drug can be targeted specifically and sufficiently to cancer cells. Numerous studies have explored the role of selected targets on the influence of ^10^B transport to make progress in developing novel, safe and effective ^10^B carriers. These findings would expand the clinical BNCT services to more patients, with potential to make BNCT a more precise and effective treatment modality for various types of cancer. This review summarizes the current knowledge of a variety of potential targets for ^10^B transport in BNCT.

## 1 Introduction

BNCT has proven to be an effective treatment for a range of cancers such as malignant glioma (1), melanoma (2), and head and neck cancer (3), confirming BNCT a promising radiotherapy for precise cancer treatment (4, 5). There are advanced features with BNCT: (i) the capability to treat recurrent tumors at sites where radiation has already been delivered, (ii) treatment of diffuse tumors where cell targeting offers potential benefit to spare healthy tissue, (iii) treatment of radiation resistant tumors by the high LET of the fission products which have potential to be more damaging than conventional radiotherapy.

General requirements for ^10^B accumulation are tumor concentrations ≥ 20 μg/g and a tumor/normal(T/N) tissue and tumor/blood(T/B) ^10^B concentration ratios of >3:1 (6). Boron delivery agents should also satisfy essential criteria: low intrinsic cytotoxicity, minimal accumulation in normal tissues adjacent to the tumor irradiation area, and effective retention of ^10^B inside cancer cells (5–7). Thus far, only two ^10^B carriers, sodium mercaptoundecahydrododecaborate-^10^B (sodium borocaptate-^10^B, BSH, Na_2_^10^B_12_H_11_SH) and boronophenylalanine-^10^B (BPA, C_9_H_12_^10^BNO_4_), have been approved for clinical BNCT (6, 8). BPA, structurally similar to phenylalanine, is primarily recognized by L-type amino acid transporter 1 (LAT1) which forms a complex with CD98 and mediates transport of BPA (9), while BSH is difficult to selectively internalize in tumor cells and accumulates in tumor through a passive diffusion (10). It is recognized there opportunity to improve upon ^10^B uptake concentration and micro-distribution offered by BPA and BSH (11, 12), with many studies exploring the role of selected targets on the influence of ^10^B delivery, and downstream therapeutic effect, towards identifying potential targets and biomarkers for BNCT (**Table 1**) (12). In this review, the primary inclusion criterion was demonstrated selective accumulation of a ^10^B-containing agent in tumor cells or tissues, regardless of whether BNCT (neutron irradiation) was subsequently performed. This approach was intended to provide a focused yet up-to-date overview of potential molecular targets influencing ^10^B transport.

**Table 1:** Targets for boron transportation in BNCT.

Target	Uptake/transport mechanism	Boron carrier	Study type	BNCT	Ref
LAT1	LAT1/CD98 mediates a Na+ and pH independent antiport of amino acids.	LAT1-overexpressing clones, BCH: a LAT1 inhibitor	*In vitro*	Yes	(13, 21)
LAT1	Preloading L−DOPA (structurally similar to L−tyrosine and BPA) enhances the antiport mechanism of BPA transporters.	L-DOPA	*In vitro* and *in vivo*	No	(11)
LAT1	Reducing intracellular efflux.	PVA-BPA	*In vitro* and *in vivo*	Yes	(26)
LAT1	High-dose L-phenylalanine reduced the accumulation of L-BPA in the normal brain relative to tumor tissue.	L-phenylalanine	*In vivo*	No	(27)
LAT2	N/A	N/A	*In vitro*	No	(9)
ATB^0,+^	ATB^0,+^ transports all amino acids except glutamate and aspartate by using a Na+ and Cl gradient	N/A	*In vitro*	No	(9)
HIF-1α	HIF-1α suppresses LAT1 expression in hypoxic cells.	YC-1	*In vitro* and *in vivo*	Yes	(40, 41)
p53	N/A	N/A	*In vitro* and *in vivo*	Yes	(38, 48, 49)
mTORC1	By inhibiting mTORC1 (a downstream target of LAT1), rapamycin reduces tumor cell proliferation and thus ^10^B loading.	Rapamycin	*In vitro*	Yes	(56, 59)
EGFR	Ligand receptor reaction.	Z33-DB, B-ASOs	*In vitro*	Yes	(7, 73)
HER2	Tetrazine moieties, attached to the surface of small boron-rich nanoparticles, could undergo the click-reaction with trans-cyclooctene mAb	Tetrazine-functionalized boron-rich carbon dots	*In vitro* and *in vivo*	No	(76)
HER2	The DARPin-modified liposomes can bind to HER2-overexpressing cells and undergo effective internalization into the cytoplasm	DARPin-modified liposomes	*In vitro*	No	(75)
Anxa1	Membrane−expressed Anxa1 mediates active transport of IF7−^10^B into both tumor vascular endothelial and tumor cells	IF7-BPA/BSH	*In vivo*	Yes	(83)
CD44	CD44 protein and translational machinery proteins as a major cell surface target and intracellular targets of BSH-polyR, respectively.	BSH-polyR	*In vitro*	No	(84)
CD44	Ligand receptor reaction.	HA/CBP-H complex	*In vitro*	Yes	(85)
TSPO	Binding at the TSPO.	1,2-closo-carboranylpyrazolopyrimidine,	*In vitro*	No	(88)
TSPO	Binding at the TSPO.	DPA-BSTPG	*In vitro* and *in vivo*	Yes	(90)
Integrin αvβ3	Cyclic RGD (cRGD) peptide-conjugated boronated albumin can directly toward integrin αvβ3	cRGD-MID-BSA, cRGD-MID-AC	*In vitro* and *in vivo*	Yes	(93)
FRα	N/A	PBC, PBC-IP	*In vitro* and *in vivo*	Yes	(99, 100)
LDLR	These nanoparticles were selectively internalized via LDLR-mediated endocytosis.	B_4_C nanoparticles	*In vitro*	No	(105)
GLUT1	Binding at the GLUT1.	Ortho-carboranylmethyl-bearing glucoconjugates	*In vitro*	No	(108)
GLUT1	Binding at the GLUT1.	6-deoxy-6-thio-carboranyl d-glucoconjugates	*In vitro*	No	(109)
GLUT1	Binding at the GLUT1.	Carboranylmethyl derivatives of d-Man, d-All, and d-Gal	*In vitro*	No	(110)
GLUT1	Binding at the GLUT1.	B-Glc	*In vitro* and *in vivo*	No	(111)
ASCT2	GluB-2 are selectively transported by ASCT2.	GluB-2	*In vitro* and *in vivo*	Yes	(113)

## 2 L-type amino acid transporter 1 and its regulation

### 2.1 L-type amino acid transporter 1

Among several amino acid transporters in cells, LAT1 (SLC7A5) is a neutral amino acid carrier highly overexpressed in tumor cells and serves as the primary mediator of BPA transportation (13). LAT1 belongs to the amino acid−polyamine−organocation superfamily and forms a heterodimeric complex with CD98 (SLC3A2) via a conserved disulfide bond. This complex mediates essential amino acid uptake in key barriers such as the placenta and blood−brain barrier (14). Studies in intact cells have shown that the LAT1/CD98 heterodimer operates a Na^+^− and pH−independent amino acid antiport (15). Functional assays using proteoliposomes reconstituted with recombinant human LAT1 identified histidine as its optimal substrate. Meanwhile, CD98 is dispensable for transport activity and presumably acts to traffic LAT1 to the cell surface (16). LAT1 is closely implicated in multiple human disorders(such as neurological diseases and malignancies) and correlates with tumor proliferation, angiogenesis, immune regulation and unfavorable clinical prognosis (17). Accordingly, LAT1, along with other nutrient transporters, has emerged as an important pharmacological target for therapeutic development (15). LAT1 expression is also regulated by multiple factors, hypoxia alters LAT1 levels via HIF−2α in differentiated neurons (18), while protein kinase C activation promotes robust endocytosis and subsequent degradation of LAT1 in HeLa cells (19).

Previous studies have assessed the relationship between LAT1 expression levels and cellular BPA accumulation. By employing genetically engineered cell lines with LAT1 knockdown or overexpression, Watanabe et al. reported that BPA uptake positively correlates with LAT1 expression (13). By analyzing data from The Cancer Genome Atlas, the relative expression rank of LAT1 in human tumor tissues is malignant melanoma, head and neck tumors, esophageal tumors, squamous cell carcinoma of the lung, cervical tumors, glioblastoma, transitional epithelial carcinoma (such as bladder and urinary tract cancer), adenocarcinoma (such as gastrointestinal, breast and uterine cancer and lung adenocarcinoma) and lymphomas. According to histological type, squamous cell carcinoma was the most common tumor with a high expression of LAT1. Elevated LAT1 expression in tumors correlates with increased proliferation marker levels and unfavorable clinical prognosis (13). In another study, intracellular uptake of BPA was 1.5–5.0 times greater among LAT1-overexpressing clones than that in a control clone, and the LAT1-overexpressing clones and transiently LAT1-transfected cells showed significant improved sensitivity to BNCT (20). In the hypoxic microenvironment, CD133−positive cells exhibit cancer stem cell characteristics, with LAT1 selectively overexpressed in these stem cell−like cells. Tani et al. constructed plasmids to induce tdTomato−tagged LAT1 overexpression on the plasma membrane of CD133−expressing cancer cells (21). Overexpressing LAT1-tdTomato in tumor improved the BPA uptake and efficacy of BNCT (21).

As LAT1 forms a complex with CD98 and mediates transport of L-type amino acid (22), several molecules structurally similar to L-type amino acids or BPA have been investigated. Multiple studies have verified that L-tyrosine treatment elevates intracellular BPA accumulation in tumor cells (23, 24). Similarly, pretreatment with substrates recognized by L or A amino acid transport systems effectively promotes BPA enrichment in gliosarcoma cells (11). These evidence support that such transporters operate via a substrate-coupled antiport exchange mechanism, which can be further activated by preloading specific amino acids. L-3,4-dihydroxyphenylalanine (L-DOPA) shares high structural similarity with L-tyrosine and BPA. Pretreament with L-DOPA can markedly boost intracellular BPA accumulation by accelerating the L-type antiport process (11, 24, 25).

LAT1 mediates BPA influx and efflux of intracellular substrates such as glutamine. When extracellular BPA is low, intracellular BPA may be exchanged for extracellular substrates like tyrosine. Poly(vinyl alcohol) (PVA), a biocompatible polymer, forms reversible boronate esters with BPA in aqueous solution. This complexation does not alter the phenylalanine structure of BPA, allowing continued recognition by LAT1. The PVA-BPA complex is internalized via LAT1-mediated endocytosis and sequestered in endo-/lysosomes, where it avoids antiport-mediated efflux by membrane-bound LAT1, thereby enhancing intracellular retention. In summary, PVA-BPA exhibited efficient accumulation and prolonged retention in tumors with quick clearance from the bloodstream and normal organs (26).

The tumor to normal tissue ^10^B concentration ratio (T/N ratio) is often used to evaluate the effect of ^10^B carriers. Instead of enhancing tumor uptake of ^10^B, decreasing the relative accumulation of ^10^B in normal tissue can be a useful way to increase the T/N ratio. Decreasing the relative accumulation of ^10^B in normal tissue may reduce the radiation-induced damage to the healthy tissue. To reduce the accumulation of ^10^B in the normal brain relative to tumor tissue, Watanabe et al. examined the effects of oral preloading with various analogues of BPA in a xenograft tumor model and found that high-dose L-phenylalanine reduced the accumulation of ^10^B in the normal brain. The L−phenylalanine group showed a 19.2% reduction in maximum irradiation dose to the normal brain, offering a simple strategy to enhance the therapeutic efficacy of conventional ^10^B agents for BNCT of brain tumors (27).

Regarding other members of LAT, Wongthai et al. reported that LAT2 can transport BPA whereas LAT3 and LAT4 cannot (9). LAT2 is mainly distributed in normal tissues, and expressed sequence tag analysis has revealed a negative correlation between LAT2 expression and tumor tissues (28, 29). Accordingly, LAT2 is presumed to modulate BPA pharmacokinetics by reducing the tumor-to-normal tissue accumulation ratio of BPA.

### 2.2 Amino acid transporter B^0,+^

Amino acid transporter B^0,+^ (ATB^0,+^), also known as solute carrier family 6 member 14 (SLC6A14), is upregulated in solid tumors and correlated with several pathological states (30). ATB^0,+^ transports most amino acids (excluding glutamate and aspartate) via Na^+^/Cl gradients (31). Its broad substrate selectivity makes ATB^0,+^ a promising candidate for targeted drug delivery applications (30, 32). A study screened aromatic amino acid transporters and found ATB^0,+^ was a transporter of BPA depending on its concentration. At 100 μM, BPA uptake was Na^+^-independent and positively linked to LAT1 protein levels, indicating LAT1 as a major transporter for BPA. At 1000 μM BPA, ATB^0,+^ measurably transports BPA (9). At a clinical dose of 250 mg BPA/kg, blood BPA reaches 2000 μM, suggesting that ATB0,+ may contribute significantly to BPA uptake in tumors expressing this transporter (33).

### 2.3 Hypoxia-inducible factor

Tumors develop hypoxic regions characterized by inadequate oxygen supply due to abnormal microvasculature via angiogenesis and rapid growth leading to insufficient blood flow (34). In hypoxic tumor cells, hypoxia-inducible factor 1 (HIF-1) accumulates as an adaptive response to hypoxia. HIF-1 is a heterodimeric transcription factor that comprises HIF-1α and HIF-1β subunits and mediates the cellular hypoxic response (35, 36). HIF-1α is pivotal in regulating tumor progression, metabolic reprogramming, excessive angiogenesis, invasive capacity and therapeutic resistance (37). Accumulated evidence has indicated that hypoxia suppresses cellular uptake of BPA and BSH across multiple cell lines (38, 39). Harada et al. further found that the hypoxia mimetic DFO markedly reduced BPA transportation, whereas the HIF-1α inhibitor YC-1 could sensitize hypoxic tumor cells to BNCT treatment (40).

Sanada et al. demonstrated that hypoxia elevated post−irradiation survival of BPA-treated squamous cell carcinoma (SCC VII) cells by down-regulating the LAT1 expression and inhibiting BPA uptake (41). In HIF−1α−deficient cells, hypoxia failed to alter the BNCT sensitivity of BPA-treated cells and LAT1 expression was not significantly reduced in hypoxia-induced cells, suggesting that hypoxia affect the sensitivity in a HIF-1α dependent manner. Furthermore, hypoxia and HIF−1α did not influence the sensitivity of BSH−treated SCC VII cells. While Masunaga et al. reported that hypoxia reduce the sensitivity of both BPA and BSH treated squamous cell carcinoma cell line to BNCT, more cells survived in the BSH group than BPA group (38). The reason may be hypoxia and HIF-1 α affect the post-irradiation survival of BPA-treated cells more than BSH-treated cells. The underlying mechanism between HIF and LAT1 has been investigated. HIF−2α upregulates LAT1 transcription by binding to its proximal promoter region, whereas direct binding of HIF−1α to the LAT1 promoter has not been detected, implying that HIF−1α does not activate LAT1 gene transcription (42). The mechanism between HIF-1α and LAT1, and the value of HIF as potential target or marker of BNCT need further investigation (41).

### 2.4 P53

The p53 gene is broadly involved in both cancer progression and suppression, as well as in cellular responses to various stressors, including hypoxia, viral infection, metabolic stress, endoplasmic reticulum stress and oxidative stress (43). p53 maintains genomic stability at G1 and G2/M cell cycle checkpoints, and regulates DNA repair and apoptosis (44, 45). p53 mutations occur frequently in most human solid tumors and regulate cellular responses to DNA−damaging therapies (e.g., radiation, chemotherapy) and hypoxic stress (45). Hypoxic stress triggers the accumulation of p53 protein and induces p53-dependent apoptosis, yet it fails to elicit p53-mediated cell cycle arrest (46). Loss of p53 function leads to resistance against DNA-damaging agents, including ionizing radiation and hypoxic stress (47). Therefore, the genetic and functional status of the p53 gene constitutes a critical factor in cancer therapeutic strategies. Wild-type p53 tumors show higher ¹^0^B accumulation than p53-mutant tumors following BPA and BSH administration (48, 49). This phenomenon is partially attributed to the higher cellular density in SAS/neo tumors compared with p53-mutant counterparts (50). Another possible explanation is that tumor cells carrying dominant-negative p53 evade genomic stability checkpoint regulation. These cells proliferate continuously without cell cycle arrest or apoptosis, thereby altering cell growth status and ultimately influencing intracellular ^10^B accumulation efficiency (43, 51).The intratumor distribution of BPA depended on the oxygen pressure in tumor tissues of p53 wild type tumors, whereas BPA uptake capacity was not dependent on oxygen pressure for p53-mutated tumor cells (38).

### 2.5 Mammalian target of rapamycin complex 1

The mechanistic target of rapamycin (mTOR) is a central regulator of cellular homeostasis and includes two complexes: mTORC1 and mTORC2. mTOR controls translation of mRNAs involved in cell cycle and proliferation (52). mTOR is a key anticancer drug target, and its inhibitors are used in combination chemotherapy (53). Rapamycin specifically inhibits mTORC1, suppresses angiogenesis, and induces autophagy (54, 55). Tatebe et al. found that rapamycin reduced the efficacy of BPA−BNCT, likely because mTOR inhibition slows tumor cell proliferation, thereby decreasing ¹^0^B loading and antitumor effects (56). Previous studies demonstrated a positive correction between LAT1 and mTORC1. Milkereit et al. reported that LAT1 in lysosomal membranes induce mTOR activation on the lysosomal membranes for the activation of mTORC1 recruited onto the lysosomal membrane (14). LAT1 facilitates mTORC1 activity by supplying cancer cells with leucine that triggers mTORC1 to regulate cancer cell growth (57–59). Inhibitors of LAT1 and knockdown of LAT1 reduced the phosphorylation of p70 S6 kinase and 4E-BP1, downstream targets of mTORC1 (60–62). These results suggest that mTORC1 may be a synergistic biomarker to predict the effect of BPA-BNCT. The underlying role of mTORC1 on BPA transportation, intracellular location and retention need further study.

## 3 Other potential targets

### 3.1 Erb-B2 receptor tyrosine kinase family

The Erb-B2 receptor tyrosine kinase family, including epidermal growth factor receptor (EGFR) and human epidermal growth factor receptor 2 (HER2), is overexpressed in various human cancers and has emerged as promising targets for BNCT.

#### 3.1.1 Epidermal growth factor receptor

EGFR, overexpressed in various human cancers and involved in angiogenesis, metastasis, and apoptosis, is considered a potential target for cancer therapy (63). EGFR is also a macropinocytosis-inducing receptor that can induce macropinocytotic cellular uptake pathways which are accompanied by clathrin-independent and actin-dependent plasma membrane ruffling and engulfment of extracellular fluids (64–67). Z33 peptide has been applied for drug delivery systems as it can specifically and effectively bind to an Fc (fragment crystallizable region) of human IgG1. A dodecaborate conjugated to the Z33 peptide (Z33‐DB) could bind to the Fc of the objective antibody, EGFR activation by EGF induced macropinocytosis, and lead to efficient cellular uptake of Z33-DB (7). To avoid EGFR conjugation problems, Rondina et al. established a computational workflow based on cetuximab (68). Cetuximab, a chimeric monoclonal antibody (mAb) for certain cancers (e.g., metastatic colorectal, lung, head/neck), has high T/B, high T/N, and low intrinsic toxicity (68). In another study, the ^10^B-rich EGFR-inhibitor, hybrid 1, was developed as a potential drug for BNCT. Hybrid 1 concentrated ^10^B in glioma cells more selectively than BPA and demonstrated a 1.2- and 1.5-times enhancement of antitumor effect of BNCT compared to BPA in glioma F98 and U87 cells, respectively (69).

Couto et al. reported that sunitinib decorated with a ^10^B cluster against glioma cancer cells was found to be 4 times more cytotoxic than sunitinib and 1.7 times more effective than BPA fructose complex when the cells were irradiated with neutrons (70). This conjugate selectively increased ¹^0^B accumulation in EGFR−overexpressing F98 glioma cells compared to normal mouse astrocytes. Hybrid erlotinib−^10^B clusters were more effective against EGFR−overexpressing cancer cells than unmodified erlotinib, with metallacarborane derivatives showing the highest activity (71). Kaniowski et al. developed antisense oligonucleotide−^10^B cluster composites as functional nanoparticles that downregulate EGFR expression and can enter EGFR−overexpressing cancer cells without a transfection agent (72). They also designed ^10^B−cluster−decorated antisense oligonucleotides (B−ASOs) targeting EGFR mRNA and confirmed that EGFR expression levels correlated with B−ASO uptake, offering higher tumor selectivity. Antisense−mediated EGFR reduction decreased ^10^B accumulation, indicating that B−ASO uptake is EGFR−dependent and represents a novel strategy for delivering therapeutic nucleic acids (and potentially other drugs) conjugated to ^10^B clusters (73).

#### 3.1.2 Human epidermal growth factor receptor 2

Human epidermal growth factor receptor 2 (HER2) overexpression is associated with aggressive tumor behavior and poor prognosis, making it an attractive target for targeted cancer therapy and BNCT (74). Proshkina et al. used HER2-specific designed ankyrin repeat protein (DARPin)_9–29 as a vector molecule on the outer surface of liposomes. These DARPin-modified liposomes were loaded with the 4-L-¹^0^BPA-D−fructose complex as the ^10^B delivery agent, which was shown to bind to HER2-overexpressing cells and undergo effective internalization into the cytoplasm *in vitro* (75). Feiner et al. proposed that tetrazine moieties, attached to the surface of small ^10^B-rich nanoparticles (NPs), could undergo the click-reaction with trans-cyclooctene mAb (TCO-mAb) (76). This would give a base for the pre-targeting approach that would lead to the desired selectivity of ^10^B rich NPs and provide a valuable alternative to current nanomaterial-based BNCT agents. They used tetrazine‐functionalized ^10^B‐rich carbon dots as a potential BNCT agent. Trastuzumab is a well-known FDA-approved humanized mAb with high target specificity and binding affinity to HER2. Pre-targeting with trans-cyclooctene-modified trastuzumab remarkably boosted intracellular ^10^B accumulation. Meanwhile, ^10^B carbon dots were found to undergo rapid *in vivo* clearance and display poor tumor enrichment following intravenous injection in a HER2-positive murine tumor model. This pretargeting strategy can improve the selectivity of tumor-targeted ^10^B delivery, thereby potentially attenuating off-tumor toxicity to the liver, lung and spleen, while elevating ^10^B accumulation within tumor tissues. Using of a pre-targeting strategy improved the accumulation of ^10^B-rich carbon dots in the tumor, with potential to improve the efficacy of BNCT (12).

### 3.2 Annexin A1

Annexin family proteins localize on the surface of endothelial caveolae and can be internalized via endocytosis (77, 78). Annexin A1 (Anxa1) plays important role in tumor growth and is a promising target for tumor vasculature (79–81). Specific Anxa1-binding carbohydrate mimetic peptide IFLLWQR (IF7) peptide effectively delivers anticancer drugs to tumors (82). For IF7-^10^B drug, membrane-expressed Anxa1 mediates the active uptake of IF7-^10^B into both tumor vascular endothelial cells and tumor cells, thereby elevating intracellular ^10^B accumulation. Neutron irradiation efficiently eliminated IF7-^10^B-treated tumor cells, while causing no obvious damage to tumor blood vessels. Neutron irradiation effectively killed cancer cells treated with IF7−^10^B but did not affect tumor vasculature. Moreover, BNCT upregulates Anxa1 in tumor tissue via inflammation or induced immunogenicity, leading to greater ^10^B accumulation after a second IF7−^10^B injection. IF7−^10^B−mediated BNCT may destroy Anxa1−positive tumor vasculature, increasing therapeutic potential. Therefore, multiple ultralow doses may be required to maximize efficacy (83).

### 3.3 Cluster of differentiation 44

Cluster of differentiation 44 (CD44), a stem cell-associated marker in various cancer types, is essential for the cellular uptake of polyR-conjugated molecules. Additionally, BSH conjugated with poly-arginine peptides has been developed (84). PolyR enhances BSH’s cell permeability and tumor selectivity, with CD44 as the main surface target and translational machinery proteins as intracellular targets. After delivery, BSH−polyR interacted with translational components, including initiation factors, termination factors, and poly(A)−binding protein. Furthermore, BSH-polyR successfully induced BNCT-dependent cell death specifically in CD44 high-expressed cells (84). Yamana et al. used sodium hyaluronate (HA), a major cellular matrix component and tumor-targeting ligand due to its high affinity for CD44 overexpressed on tumor cell surfaces, as a nanocarrier building block to prepare a water-soluble hyaluronic acid/pyrene-substituted o-carborane (HA/CBP-H) complex. After thermal neutron irradiation, this complex produced excellent cytotoxicity, equal to or greater than that of the clinically-used BPA-fructose (85).

### 3.4 Translocator protein

The translocator protein (TSPO) is an 18 kDa protein with five-transmembrane domain of 169 amino acid residues located in the outer mitochondrial membrane of cells in a variety of tissues (86, 87). Crossley et al. reported a new, ^10^B-rich carboranylpyrazolopyrimidines, 1,2-closo-carboranylpyrazolopyrimidine, which possesses a unique two-site binding profile at the TSPO and both closo- and nido-carborane derivatives are able to accumulate in T98G human glioma cells at about 13 times higher than BPA and 14 times higher than BSH (88). A ^10^B compound targeting TSPO, closo-dodecaborate [(B_12_H_11_)^2-^] anion-containing translocator protein ligand, significantly improved the ^10^B accumulation through convection-enhanced delivery administration compared to BPA intravenous administration in the F98 rat glioma bearing brain tumor model. BNCT using DPA-BSTPG demonstrated more efficacy over the untreated group. Combined BNCT with BPA and DPA−BSTPG achieved markedly prolonged survival compared with BPA monotherapy (89, 90).

### 3.5 Integrin αvβ3

Serum albumin is an abundant protein that has an extraordinary ligand-binding capacity to carry various endogenous and exogenous compounds in plasma (91). The serum albumin accumulates in tumor due to the combination of leaky and abnormal blood vessels with the absence or defect of the lymphatic drainage system known as the enhanced permeability and retention effect (92). Cyclic RGD (cRGD) peptide-conjugated boronated albumin was developed to direct toward integrin αvβ3, which overexpresses on many cancer cells. A stepwise conjugation of c[RGDfK(Mal)] and maleimide-conjugated closo-dodecaborate (MID) to bovine serum albumin (BSA) afforded cRGD-MID-BSA, which was noncytotoxic to cancer cells. In both *in vitro* and *in vivo* models, cRGD-MID-BSA exhibited superior selective accumulation, prolonged retention, and stronger antitumor activity against integrin αvβ3-overexpressing U87MG cells relative to BPA (93). Previously, MID albumin conjugate (MID-AC) was found as an effective ^10^B carrier. Recently, Kawabata et al. developed cRGD-functionalized MID-AC which is a αvβ3-targeted long-retention-type ^10^B carrier and highly selective against gliomas (93, 94).

### 3.6 Folate receptor α

Folate receptors are cell membrane proteins responsible for binding folate, a B−vitamin essential for DNA synthesis. Folate receptor α (FRα) is important in embryogenesis but minimally expressed after birth; however, it is overexpressed in many solid tumors while low in healthy tissues (95). Accordingly, FRα is significantly upregulated in malignant gliomas than in normal tissues (96, 97). Water−soluble pteroyl−closo−dodecaborate conjugates (PBCs) were developed for BNCT, showing low cytotoxicity and selective accumulation in FRα−positive cells (e.g., U87MG), but their *in vivo* tumor accumulation was limited by poor blood retention (98, 99). To overcome this limitation, the research group synthesized PBC−IP, a pteroyl−closo−dodecaborate derivative conjugated with 4−(p−iodophenyl)butyric acid (100). This molecule integrates three functional domains: an FRα targeting motif, a ^10^B carrier containing twelve ^10^B atoms, and an albumin−binding fragment. Conjugation with an albumin ligand enables PBC derivatives to bind endogenous albumin, prolonging circulatory retention and facilitating tumor enrichment (101, 102). PBC−IP was selectively taken up by C6, F98, and U87MG glioma cells, achieving higher ^10^B accumulation than BPA both *in vitro* and *in vivo*. When administered via convection−enhanced delivery (CED) in an F98 orthotopic glioma model, PBC−IP produced T/N and T/B ^10^B ratios of 37.8 and 94.6, respectively, at 3 h post−CED. Post−BNCT survival at 180 days was 50% with PBC−IP alone and 70% with combined BPA and PBC−IP, with no residual tumors. Unlike BPA, which requires fructose/sorbitol for solubility, PBC−IP is water−soluble without additives. CED allows approximately 100−fold dose reduction of PBC−IP compared to BPA, thereby minimizing adverse effects such as BPA−induced crystalluria (100). It should be noted that CED is technically complex and invasive, with unproven clinical benefit, and remains largely an investigational technique (103).

### 3.7 Low-density lipoprotein receptor

Low-density lipoprotein receptor (LDLR) is a transmembrane glycoprotein that plays a central role in cholesterol homeostasis by mediating the cellular uptake of low-density lipoproteins via receptor-mediated endocytosis. LDLR is overexpressed in various cancers, including gliomas, hepatocellular carcinoma, prostate cancer, lung cancer, breast cancer, and colorectal cancer, due to the increased demand for cholesterol and lipids required for rapid tumor cell proliferation (104). Rudawska et al. developed functionalized ^10^B carbide (B_4_C) nanoparticles as active ^10^B delivery agents for BNCT. These nanoparticles were functionalized with anti-LDLR antibodies to achieve active targeting of LDLR-overexpressing cancer cells, and they were selectively internalized via LDLR-mediated endocytosis. *In vitro* studies demonstrated that the B_4_C anti-LDLR nanoparticles achieved a ^10^B concentration of 9.58 ± 2.6 mg/L per million cells in SCC-25 cells (105).

### 3.8 Glucose transporter 1

Glucose transporter 1 (GLUT1) is a facilitative glucose transporter that mediates the uptake of glucose across the plasma membrane. GLUT1 is overexpressed in a wide range of human cancers due to the increased glucose demand associated with the Warburg effect. This distinct overexpression profile of GLUT1 in cancer cells, compared to normal tissues, renders it a highly attractive target for tumor-selective drug delivery strategies (106).

Matović et al. conducted a series of systematic studies to develop glucose-based ^10^B carriers targeting GLUT1 (107–110). Initially, a library of ortho-carboranylmethyl-bearing glucoconjugates was synthesized, and the substrate specificity of these compounds for GLUT1 was evaluated. In cellular uptake studies using CAL 27 cells (human tongue squamous cell carcinoma), all glucoconjugates delivered significantly higher ^10^B content than BPA and BSH (107). Next, the entire positional isomer library of ortho-carboranylmethyl-bearing glucoconjugates was synthesized and assessed through a comprehensive *in vitro* evaluation. The glucoconjugates were found to compete with the natural substrate for GLUT1, to deliver significantly higher ^10^B content (approximately 1.5- to 3-fold higher) to CAL 27 cells than BPA and BSH, and not to enter the common metabolic routes of D-glucose (108). Subsequently, short and accessible synthetic methods were developed for the construction of a set of 6-deoxy-6-thio-carboranyl d-glucoconjugates, and molecular recognition studies revealed that the atom involved in cluster conjugation has a marked effect on GLUT1 affinity, with species connected to a carbon atom exhibiting significantly higher affinity than those connected to a ^10^B atom (109). Since hexose transporters, including GLUT1, also recognize monosaccharides other than d-Glc, the corresponding carboranylmethyl derivatives of d-Man, d-All, and d-Gal (epimers of d-Glc) were synthesized. Using docking studies with a GLUT1 model derived from d-xylose-proton symporter crystal structures (PDB ID 4QIQ and 6N3I) and cis-inhibition assays in CAL 27 cells, glycoconjugates 1–3 displayed stronger binding affinity toward GLUT1 than d-Glc and exhibited superior glucose transporter targeting capabilities (IC_50_ values all < 1 mM) compared to the natural substrate (IC_50_ > 1 mM), although none matched the affinity of the hit compound 6-oCb-Glc (IC_50_ = 43.96 μM). The ^10^B delivery capacity of glycoconjugates 1–3 was found to be superior to that of clinically used agents BPA and BSH, with modification at position 6 emerging as a promising strategy regardless of stereochemical configuration, and all three glycoconjugates exhibited comparable ^10^B delivery efficiency to the previous hit compound 6-oCb-Glc (110). A recent study extended the *in vitro* investigations to three additional patient-derived HNSCC cell lines (UT-SCC-14, UT-SCC-28, and UT-SCC-42B) and evaluated *in vivo* pharmacokinetics in HNSCC tumor xenografts. Positron emission tomography - computed tomography (PET-CT) imaging using [ (18)F]fluoro-2-deoxy-d-glucose ([¹^8^F]FDG) radiotracer confirmed increased glucose uptake in CAL 27 and UT-SCC-14 tumors *in vivo*, supported by GLUT1 expression observed in tumor section immunohistochemistry. 6-O-(o-carboranylmethyl)-d-glucopyranose (B-Glc) demonstrated superior uptake and favorable kinetic parameters compared to BPA and BSH across all tested cell lines, with peak ^10^B accumulation at 15–30 min post-injection (75 mg/kg dose) achieving the required tumor ^10^B concentration (>20 ppm) for effective BNCT, comparable to the clinical BPA-fructose complex (400 mg/kg dose) at 60 min (111). However, neutron irradiation was not conducted in these studies, leaving BNCT efficacy unvalidated.

### 3.9 Alanine-serine-cysteine transporter 2

Alanine-serine-cysteine transporter 2 (ASCT2) is a sodium-dependent neutral amino acid transporter that mediates the cellular uptake of glutamine, alanine, serine, and cysteine. ASCT2 is overexpressed in a wide range of human cancers due to the increased demand for glutamine to support tumor cell proliferation (112). This characteristic makes ASCT2 an attractive target for tumor-selective ^10^B delivery, particularly for tumors that are refractory to L-BPA. Miura et al. developed a water-soluble, ASCT2-targeted small-molecule ¹^0^B carrier GluB-2. *In vitro* assessments across multiple cancer cell lines—especially those characterized by low LAT1 and high ASCT2 expression—revealed that GluB-2 possesses high solubility, low cytotoxicity, and selective uptake mediated by ASCT2. *In vivo* experiments demonstrated that GluB-2, administered either intravenously or intraperitoneally, achieved tumor ^10^B concentrations exceeding the therapeutic benchmark of >20 μg ¹^0^B/g tissue. Following thermal neutron irradiation, GluB-2 led to marked tumor suppression in both the CT26 allograft and the BPA-refractory U87MG xenograft models, with no observed systemic toxicity (113).

## 4 Conclusion and perspectives

BNCT represents a promising treatment modality, with data confirming the safety and efficacy of treatment for a number of tumor types. Nevertheless, clinically used BPA and BSH cannot fulfill all criteria of the treatment. As a result, significant efforts are being made to develop novel ^10^B carriers as well as optimizing the administration protocol of the currently used drugs. Unfortunately, none of the novel ^10^B carriers have yet been approved for clinical application. Similar to the development of low selectivity chemotherapy to current high selectivity immune and targeted therapy, creating a selective and effective ^10^B carrier is a very valuable development. Therefore, finding potential targets influencing ^10^B transport may be beneficial for developing innovative ^10^B carriers for BNCT. In addition, clarifying the mechanism by which these potential targets affect the import and export of ^10^B will help improve ^10^B uptake and retention. In the review, we discussed the latest research results of potential targets, their mechanisms affecting ^10^B transport, and related agents. It is hoped that through the efforts of further study, novel ^10^B carriers will expand BNCT indications, and unlock the potential of BNCT being highly effective and less toxic, benefiting cancer patients.
